# Supplementary material for: Targeting endothelin receptor signalling overcomes heterogeneity driven therapy failure
Source: EMBO Mol Med. 2017 Jun 12;9(8):1011–29. doi: 10.15252/emmm.201607156 (PMC5538298; doi:10.15252/emmm.201607156)
Supplement: Supplementary file 1 — Appendix [file EMMM-9-1011-s001.pdf]

Appendix

Appendix Figures

Appendix Figure S1, related to Figure 1
MITF and AXL expression are heterogeneous in melanoma

Appendix Figure S2, related to Figure 3
Long-term BRAFi-treatment produces drug-tolerance

Appendix Figure S3, related to Figure 4
EGFR and ECE1 expression in melanoma cells

Appendix Figure S4, related to Figure 5
EDN1 and EDNRB are involved in paracrine protection

Appendix Figure S5, related to Figure 7
EDNR inhibition overcomes paracrine protection in AXL-high cells

Appendix Table S1, related to Figure 1
Patient Characteristics

Appendix Table S2, related to Figure 7
Patient characteristics corresponding to short-term cultures

Appendix Table S3, related to Materials and Methods
Schematic of injections into zebrafish embryos

Appendix Supplementary Methods

Appendix Supplementary References

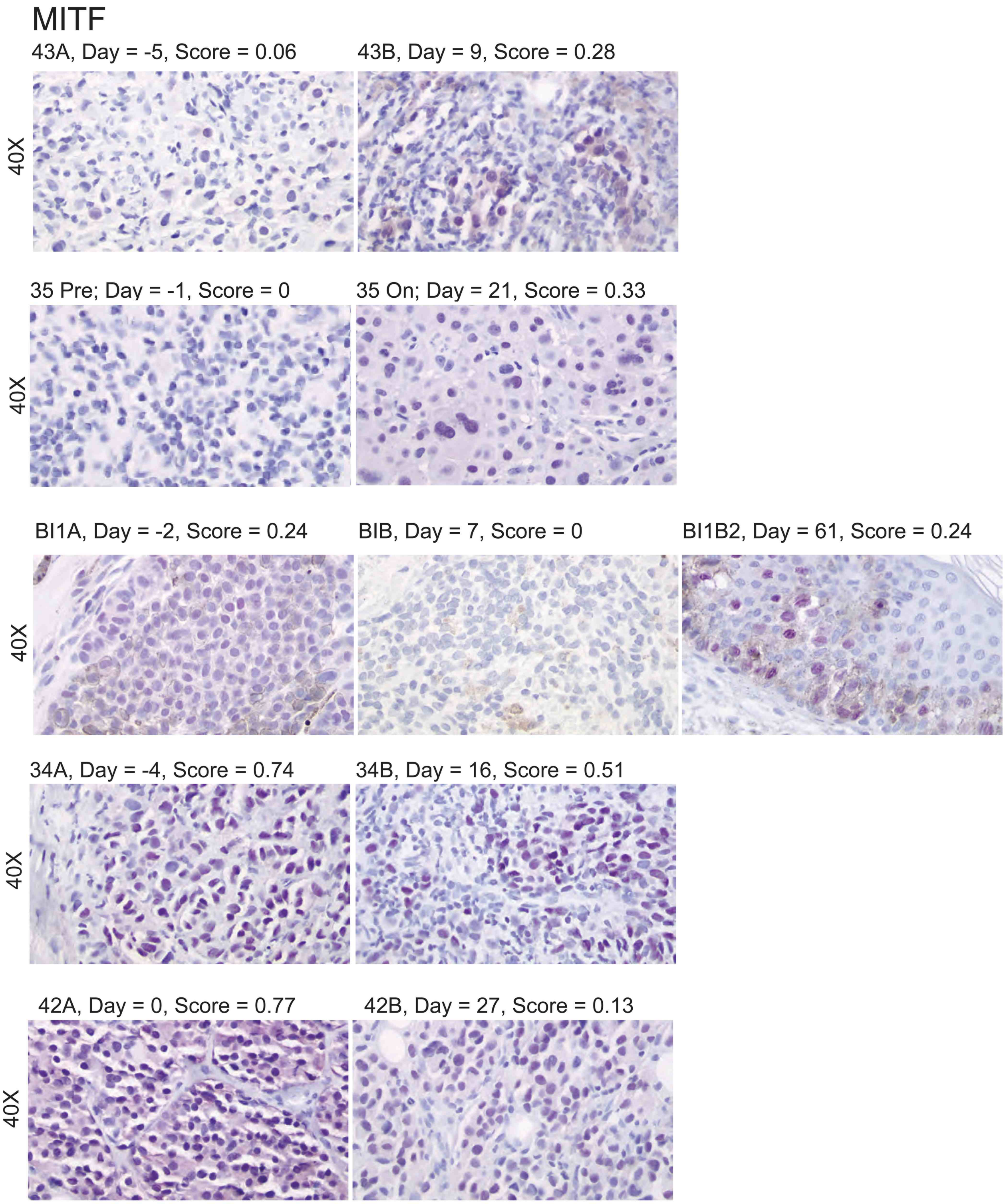

Appendix Figure S1, related to Figure 1: MITF expression on treatment is heterogeneous

IHC analysis for MITF in biopsies taken from patients before and on treatment with BRAF and MEK inhibitor for the indicated days. For patient information see Table S1.

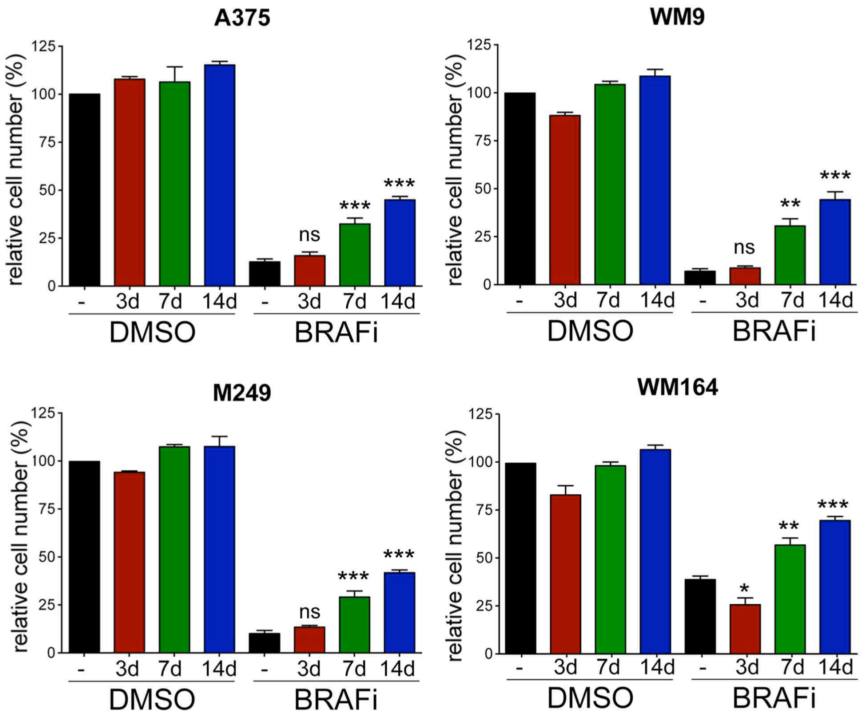

Appendix Figure S2, related to Figure 3: Long-term BRAFi-treatment produces drug-tolerance

Quantification of relative cell number of the indicated cell lines when treated with DMSO or vemurafenib (BRAFi). The respective cell lines were either naïve or had been pre-treated with BRAFi for 3, 7 or 14 days. *P*: probability by one-way ANOVA (Dunnett) $P = 0.0005$ (A375 7d), $P < 0.0001$ (A375 14d), $P = 0.0002$ (WM9 7d), $P < 0.0001$ (WM9 14d), $P = 0.0004$ (M249 7d), $P < 0.0001$ (M249 14d), $P = 0.0329$ (WM164 3d), $P = 0.00065$ (WM164 7d), and $P = 0.0002$ (WM164 14d). Data are from three independent experiments are pooled and presented as mean ±SEM. *, **, and ***: $P < 0.05$, $P < 0.01$, and $P < 0.001$, respectively.

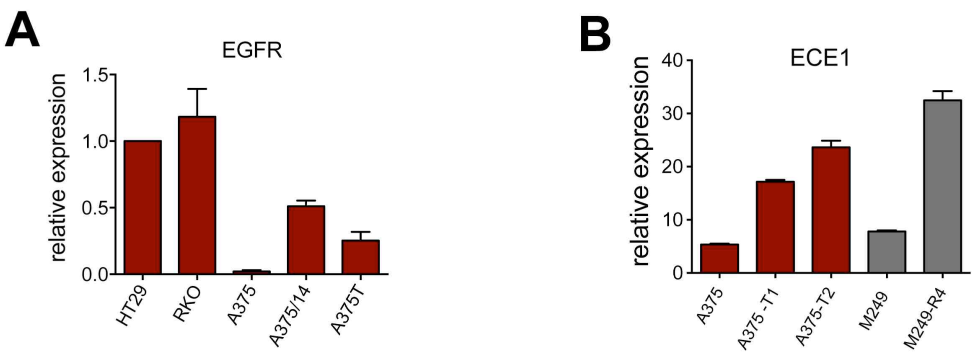

Appendix Figure S3, related to Figure 4: EGRF and ECE1 expression in melanoma cells expression is up-regulated in response to long-term MAPKi treatment

A. Real-time qPCR analysis of EGFR expression in the indicated cell lines. HT29 and RKO colorectal cancer cell lines served as positive control.

B. Real-time qPCR analysis of ECE1 expression in the indicated melanoma cell lines.

Data are the mean±SEM.

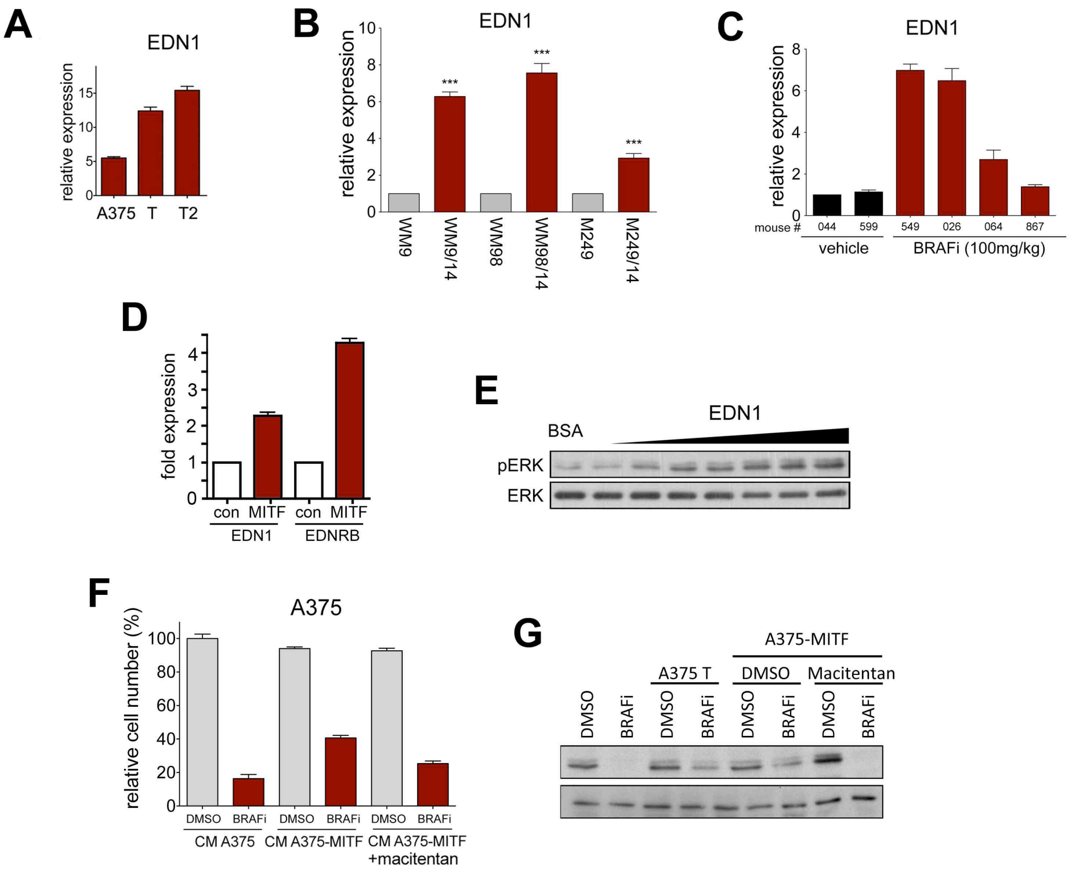

Appendix Figure S4, related to Figure 5: EDN1 and EDNRB are involved in paracrine protection

A, B Real-time qPCR analysis of EDN1 expression in the indicated cell lines. *P*: probability by one-way ANOVA (Tukey), $P < 0.0001$. Data are from three independent experiments and presented as mean ±SEM.

C. Real-time qPCR analysis of EDN1 expression in A375-GFP cells. A375-GFP cells had been isolated from xenografts grown in mice treated with either vehicle or 100mg/kg vemurafenib (BRAFi) for 12 days.

D. Real-time qPCR analysis of EDN1 and EDNRB expression in A375 cells transfected with either an empty vector control (con) or an MITF expressing plasmid.

E. Dose response of ERK phosphorylation to increasing concentrations of EDN1 (2, 4, 6, 8, 10, 20 and 40 ng/ml).

F. Relative cell number of A375 cells treated with BRAF inhibitor in the presence of conditioned medium from either A375 cells or A375 cells over-expressing MITF.

G. Western blot of A375 cells treated as indicated for pERK and ERK.

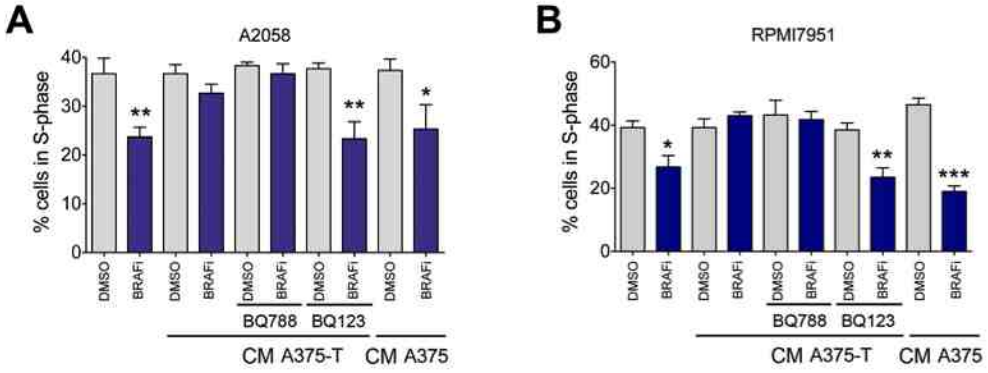

Appendix Figure S5, related to Figure 7: EDN1 and EDNRB are involved in paracrine protection

A, B Analysis of cells in S-phase. The indicated cell lines were treated with vemurafenib (BRAFi), BQ788 or BQ123 either alone or in combination and in the presence of conditioned medium from A375-T cells for 24 h, and 4 h before analysis EDU was added to the cultures. The conditioned medium from A375 cells was used as control. *P*: probability by one-way ANOVA (Dunnett) $P = 0.0093$ (A2058 BRAFi), $P = 0.0074$ (A2058 BRAFi BQ123), $P = 0.0279$ (A2058 BRAFi A375CM), $P = 0.0233$ (RPMI BRAFi), $P = 0.0025$ (RPMI BRAFi BQ123), and $P < 0.0001$ (RPMI BRAFi A375CM). Data are form three independent experiments and are presented as mean ±SEM. *, **, and ***: $P < 0.05$, $P < 0.01$, and $P < 0.001$, respectively.

Appendix Table S1: Patient Characteristics

Patient	Mutation	Treatment	Response (maximal response in %)	Time to Progression (months)
2	BRAF	vemurafenib	PR (-60.5)	8.5
3	BRAF	vemurafenib	SD (-10%)	10
4	BRAF	vemurafenib	PR (-56%)	3.5
5	BRAF	vemurafenib	SD (-27%)	6.5
6	BRAF	dabrafenib_trametinib	PR (-59.9%)	21
7	BRAF	dabrafenib_trametinib	CR (100%)	17
8	BRAF	dabrafenib & trametinib	PR (-30%)	5
9	BRAF	dabrafenib & trametinib	PR (-45%)	7
10	BRAF	dabrafenib & trametinib	SD (-13%)	3
11	BRAF	dabrafenib & trametinib	PR (-80%)	10
12	BRAF	dabrafenib & trametinib	PR (-88.9%)	12, stop at 20 months
13	BRAF	dabrafenib & trametinib	PR (-57.9%)	9, stroke
16	BRAF	dabrafenib_trametinib	SD (-19.5%)	11
18	BRAF	dabrafenib & trametinib	SD (-16.5%)	6
24	BRAF	vemurafenib	PR (-53%)	2
25	BRAF	dabrafenib & trametinib	PR (-64%)	3
34	BRAF	LGX818 +MEK 162	PR (-48.6%)	15
35	BRAF	LGX818 +MEK 162	PR (-38.6%)	10
40	BRAF	vemurafenib	PR	9
42	BRAF	LGX818 + MEK 162	PR (-76.1)	13
43	BRAF	vemurafenib	CR (100%)	44
BI1	BRAF	LGX818 +MEK 162	PR (-42.9)	30

PR = partial response SD = stable disease

Appendix Table S2: Patient Characteristics corresponding to short-term cultures

Cell culture	Treatment	Treatment Duration (days)	Mutation	Tumour Location
M121224	LGX818	115	$BRAF^{V600E}$ & $NRAS^{Q61K}$	Cutaneous
M130429	MEK162	57	$MITF^{E318K}$ & $NRAS^{Q61R}$	Bone
M130219	MEK162	21	$MITF^{E318K}$ & $NRAS^{Q61R}$	Cutaneous

Patients with metastatic melanoma harboring a ^{V600E}BRAF or ^{Q61R}NRAS mutation (confirmed by genotyping) were enrolled on clinical trials for treatment with LGX818 (BRAFi) or MEK162 (MEKi). Mutation, treatment and duration until melanoma cells were isolated from the indicated tumor location are indicated.

Appendix Table S3: Schematic of injections into zebrafish embryos

xenograft	A375 homogenous		A375-T homogenous			A375-T/A375 heterogenous	
cell line	A375	A375	A375-T	A375-T		A375-T	A375
cell number	500	500	500	500		500	500

Zebrafish xenografts were generated by injection of approximately 1000 melanoma cells in total into the space surrounding the heart of embryos 48 h post-fertilization.

Appendix Supplementary Methods

Cell lines

A375 and WM266-4 cells were bought from the American Type Culture Collection and 501mel and 888mel cells were a gift from Steve Rosenberg (NCI, MD); all were obtained in 2008. The human melanoma cell line WM164 used in the single cell analysis was genotypically characterized (1,2), grown as described (3) with 4% fetal bovine serum and authenticated by STR fingerprinting (Genomics Facility, The Wistar Institute, Philadelphia, PA; Analytical Facility, QIMR Berghofer Medical Research Institute, Herston, QLD, Australia). Additional cell lines in the panel were a gift from Meenhard Herlyn (Wistar Institute) and Adam Hurlstone (University of Manchester). All cell lines were authenticated in house by short tandem repeat profiling before and during the study; the last authentication was carried out in 2014. These cell lines were grown in DMEM/10% FCS (PAA, Yeovil, UK).

Cell culture reagents and treatments

Vemurafenib, AZD6244, PD184352, Go-6983, RAF265, dovotininb, bosentan and macitentan were from Selleck Chemicals (Newmarket, UK). BQ788 and BQ123 were from Tocris. siRNAs transfections were carried out using lipofectamine or transferrin. All siRNAs were from Dharmacon/Thermo Fisher and the sequences were: SC control: AAUAUAAUCACUAUCAGGUGC; MITF#1: GAACGAAGAAGAAGAUUUAUU; MITF#3: GACCUAACCUGUACAACAAUU, EDN1: *SMART*pool L-016692-00. For drug-dose response curves cells were plated in 96-well plates at a density of $0.1x10^5$ and treated with serial dilutions of the drugs as indicated. After treatment cells were fixed and stained with crystal violet and the concentration required to inhibit cell growth by 50% (GI50) was calculated using GraphPad Prism version 6.0a for Mac OS, GraphPad Software (San Diego California, USA).

Incucyte analysis

Apoptotic cells were detected by caspase 3/7 dependent cleavage of a florescent reagent (Essen BioScience Cat no 4440). Cells were imaged using an IncuCyte ZOOM (Essen BioScience) under 20× objective, under normal cell culture conditions. Phase contrast and fluorescence images were acquired every 20mins with four images per well. IncuCyte ZOOM software was utilized in real-time for the measurement of apoptosis, this data was further analysed in Prism 6 (GraphPad).

Co-culture analysis

Cells were plated at a density of $1x10^5$ per 6 well trans-well insert with a pore size of 0.4μm (Corning, catalogue number: 3412), and left to adhere for 24 h before being transferred to six wells with already seeded melanoma cell lines. Cells were treated with inhibitors after 24 h of co-culturing for indicated times followed by either cell lysis or cell survival analysis. Cell numbers were assessed by crystal violet staining of cells fixed in 4% formaldehyde. Quantification was achieved via spectrophotometrical analysis of the optical density at 540nM (OD 540) of the solubilized dye.

Cell lysis and immunoblotting

Cells were lysed in SDS sample buffer and analysed by standard Western-blotting protocols. The primary antibodies used were: phospho-ERK (MAPK-YT) from Sigma, St Louis, MO, USA; ERK2 (C-14) and beta-tubulin (H235) from Santa Cruz Biotechnology, USA; MITF (C5) from Fisher Scientific, UK; EDN1 mouse monoclonal Antibody was from Abcam, UK. PKC substrate (cat # 6967) from Cell Signaling.

RNA analysis and qPCR primers

RNA from cell lines or frozen tumour tissue was isolated with TRIZOL® as described previously (4,5). Selected genes were amplified using SYBR green (Qiagen, Valencia, CA,

USA). Patient samples were pre-amplified using the TaqMan PreAmp Master Mix Kit (PN4384267, Applied Biosystems, Carlsbad, CA) according to the manufacturer's instructions. TaqMan qPCR was carried out with TaqMan Gene Expression Master Mix (PN4369016, Applied Biosystems, Carlsbad, CA). Primer sequences for SYBR green qPCR were: MITF: CCGTCTCTCACTGGATTGGT, TACTTGGTGGGGTTTTCGAG; GAPDH: CAATGACCCCTT-CATTGACC, GACAAGCTTCCCGTTCTCAG; BETA-ACTIN: GCAAGCAGGAGTATGACGAG, CAAATAAAGCCATGCCAATC; EDN1: GCTCGTCCCTGATGGATAAA, TTCCTGCTTGGCAAA-AATTC; ECE1: Qiagen cat# HS_ECE1_1_SG.

Immunohistochemistry and immunofluorescence

After de-paraffinisation, antigen retrieval was performed by the pressure cooker method using EDTA buffer, pH 8.0. Staining was performed using an automated system (Autostainer plus Dako). A monoclonal mouse antibody raised against a N-terminal fragment of MITF protein of human origin (Santa Cruz Biotechnology, sc-56433) was used at a 1:60 dilution. Sections were counterstained with hematoxylin. Additional anti-bodies are listed in the table below.

Name	Species	Company	Cat No
aSMA	Mouse	Invitrogen	18-0106
AXL	Rabbit	Sigma	HPA037423
CD34	Rabbit	Abcam	ab81289
Cleaved Caspase	Rabbit	Cell Signalling	9661
ERK 1/2 phospho	Rabbit	Cell Signalling	9101
ki67	Rabbit	Abcam	ab16667
MITF D5	Mouse	Thermo	MS-772-P0

For immunofluorescence tumour samples either derived from a patient treated with dabrafenib or taken from mice treated with either vemurafenib (25mg/kg) or selumetinib (10mg/kg) were fixed and cryo-sections were permeabilised in a solution of 0.1 % Trition-X100 and 1 % saponin in PBS for 15 min. Sections were blocked in 10 % BSA at 37°C for 30 min and incubated overnight at 4°C with primary MITF antibody ((D5) Neomarkers, Lab Vision, Runcorn, UK) in 10 % BSA PBS. Stained sections were washed in PBS and then incubated with Cy3-labelled secondary antibody for 2 hr at RT and mounted using DAPI containing vectashield. For cell staining, cells were fixed with 4 % formaldehyde in PBS for 15 min before permeabilization with 0.1 % Triton-X100 and the protocolled followed as described above using the indicated antibodies. A Zeiss Axioskop 2 plus equipped with a 40x Plan Neofluar® objective was used; images were taken by a Photometrics Cool Snap HQ CCD camera driven by Metamorph software (Universal Imaging).

Mass Spectrometry and data analysis

Cells were cultured in serum-free medium for 48-72h. The supernatant was collected, cooled on ice and spun at 2000g for 20 min to remove debris and subsequently filtered through a 0.22 filter. Exosomes were removed by spinning the supernatant further at 100000g for 1 h. The samples were additionally concentrated with spin cut-off membranes with a 3-5kDa cut-off. Samples were frozen and subsequently lysed by boiling them in 1% SDS, 0.1M DTT. The FASP protocol (6) was used to remove SDS, alkylate cysteine and digest the proteins. The peptides were analysed by mass spectrometry as previously described (7). Protein identification quantification was performed by label-free quantification with the MaxQuant software package (8).

Appendix Supplemental References

1. Hoek KS, Schlegel NC, Brafford P, Sucker A, Ugurel S, Kumar R*, et al.* Metastatic potential of melanomas defined by specific gene expression profiles with no BRAF signature. Pigment cell research / sponsored by the European Society for Pigment Cell Research and the International Pigment Cell Society **2006**;19(4):290-302
2. Smalley KS, Contractor R, Haass NK, Lee JT, Nathanson KL, Medina CA*, et al.* Ki67 expression levels are a better marker of reduced melanoma growth following MEK inhibitor treatment than phospho-ERK levels. British journal of cancer **2007**;96(3):445-9
3. Smalley KS, Brafford P, Haass NK, Brandner JM, Brown E, Herlyn M. Up-regulated expression of zonula occludens protein-1 in human melanoma associates with N-cadherin and contributes to invasion and adhesion. The American journal of pathology **2005**;166(5):1541-54.
4. Smith MP, Ferguson J, Arozarena I, Hayward R, Marais R, Chapman A*, et al.* Effect of SMURF2 targeting on susceptibility to MEK inhibitors in melanoma. J Natl Cancer Inst **2013**;105(1):33-46.
5. Smith MP, Sanchez-Laorden B, O'Brien K, Brunton H, Ferguson J, Young H*, et al.* The immune microenvironment confers resistance to MAPK pathway inhibitors through macrophage-derived TNFalpha. Cancer discovery **2014**;4(10):1214-29.
6. Wisniewski JR, Zougman A, Nagaraj N, Mann M. Universal sample preparation method for proteome analysis. Nature methods **2009**;6(5):359-62.
7. Farrell J, Kelly C, Rauch J, Kida K, Garcia-Munoz A, Monsefi N*, et al.* HGF induces epithelial-to-mesenchymal transition by modulating the mammalian hippo/MST2 and ISG15 pathways. Journal of proteome research **2014**;13(6):2874-86.
8. Cox J, Hein MY, Luber CA, Paron I, Nagaraj N, Mann M. Accurate proteome-wide label-free quantification by delayed normalization and maximal peptide ratio extraction, termed MaxLFQ. Molecular & cellular proteomics : MCP **2014**;13(9):2513-26 doi 10.1074/mcp.M113.031591.
