# Supplementary material for: Targeting endothelin receptor signalling overcomes heterogeneity driven therapy failure
Source: EMBO Mol Med. 2017 Jun 12;9(8):1011–29. doi: 10.15252/emmm.201607156 (PMC5538298; doi:10.15252/emmm.201607156)
Supplement: Supplementary file 2 — Expanded View Figures PDF [file EMMM-9-1011-s002.pdf]

Expanded View Figures

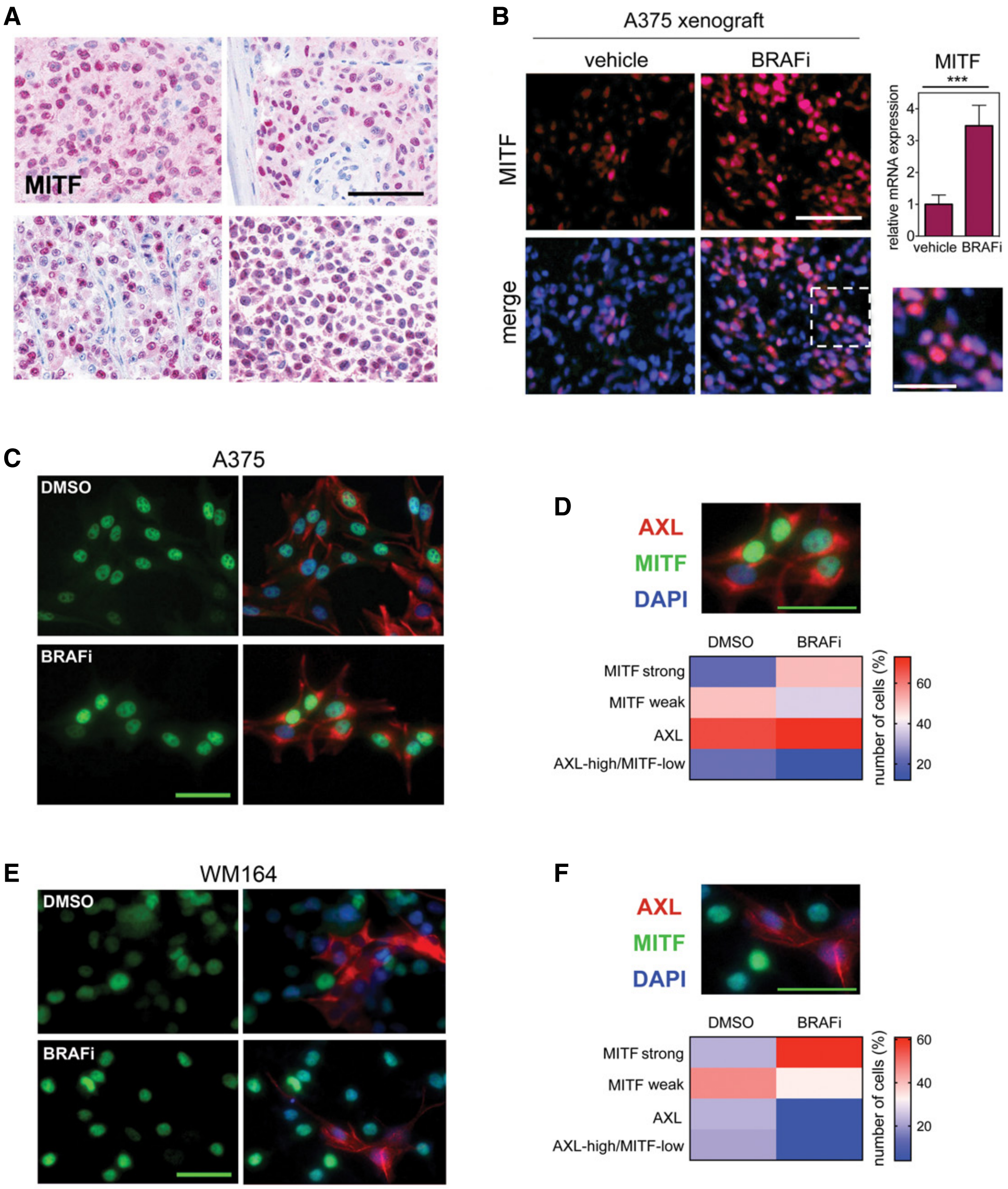

Figure EV1. MITF and AXL expression are heterogeneous in melanoma.

A Immunohistochemistry analysis of MITF expression in human melanoma specimens. Scale bar: 100 μm.

B Immunofluorescence analysis of MITF expression (magenta) in xenografts from mice treated with either vehicle or 25 mg/kg PLX4720 (BRAFi) for 3 weeks. Nuclei were stained with DAPI. Scale bar: 100 μm. Dashed line indicates origin of magnification; scale bar: 50 μm. RT-qPCR for MITF from xenografts (n = 5) is shown. *** indicates probability by t-test; $P < 0.0001$. Data are presented as mean ± SEM.

C Immunofluorescence analysis in A375 melanoma cells treated with either DMSO or vemurafenib for 3 days, before they were stained for MITF (green) and AXL (red) expression. Scale bar: 50 μm.

D Quantification of MITF and AXL co-staining in single cells. Scale bar: 50 μm.

E Immunofluorescence analysis in WM164 melanoma cells treated with either DMSO or vemurafenib for 3 days, before they were stained for MITF (green) and AXL (red) expression. Scale bar: 50 μm.

F Quantification of MITF and AXL co-staining in single cells. Scale bar: 50 μm.

© 2017 The Authors

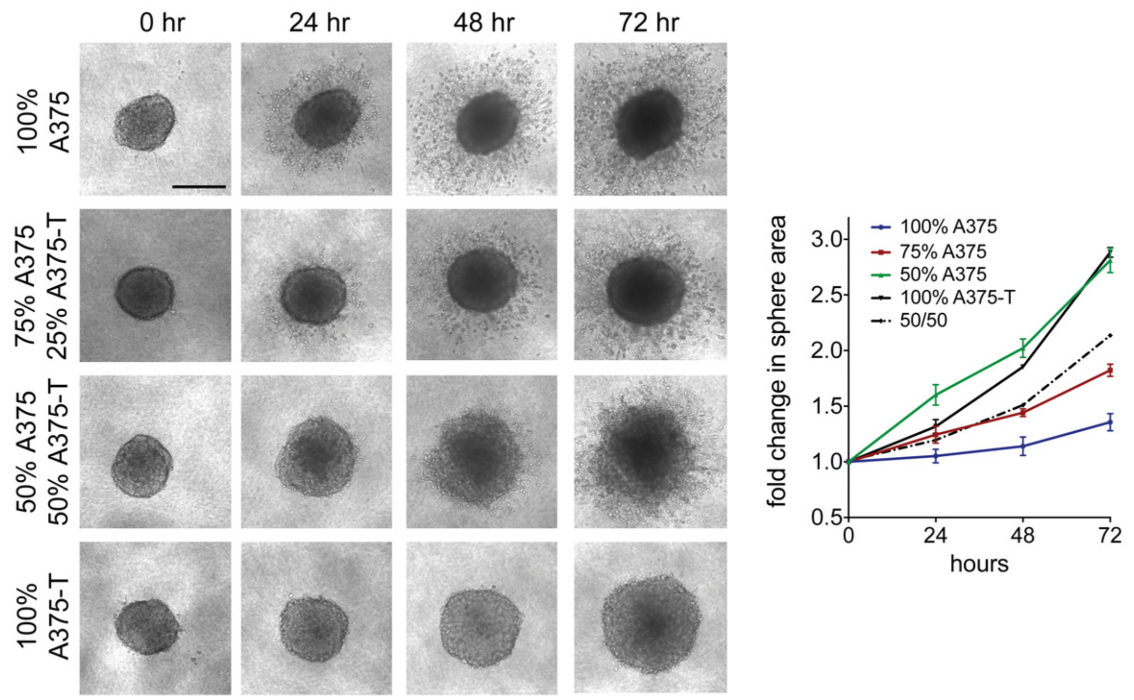

Figure EV2. Paracrine signals enhance melanoma sphere growth and invasion.

The growth of 3D collagen embedded spheres of A375 cells or A375-T cells combined at the indicated ratios was monitored over 72 h. Images were taken, and sphere areas were quantified using ImageJ. Scale bar: 200 μm. The dotted line represents the predicted growth of a 50/50% sphere. Data are pooled from three independent experiments and presented as mean ± SEM.

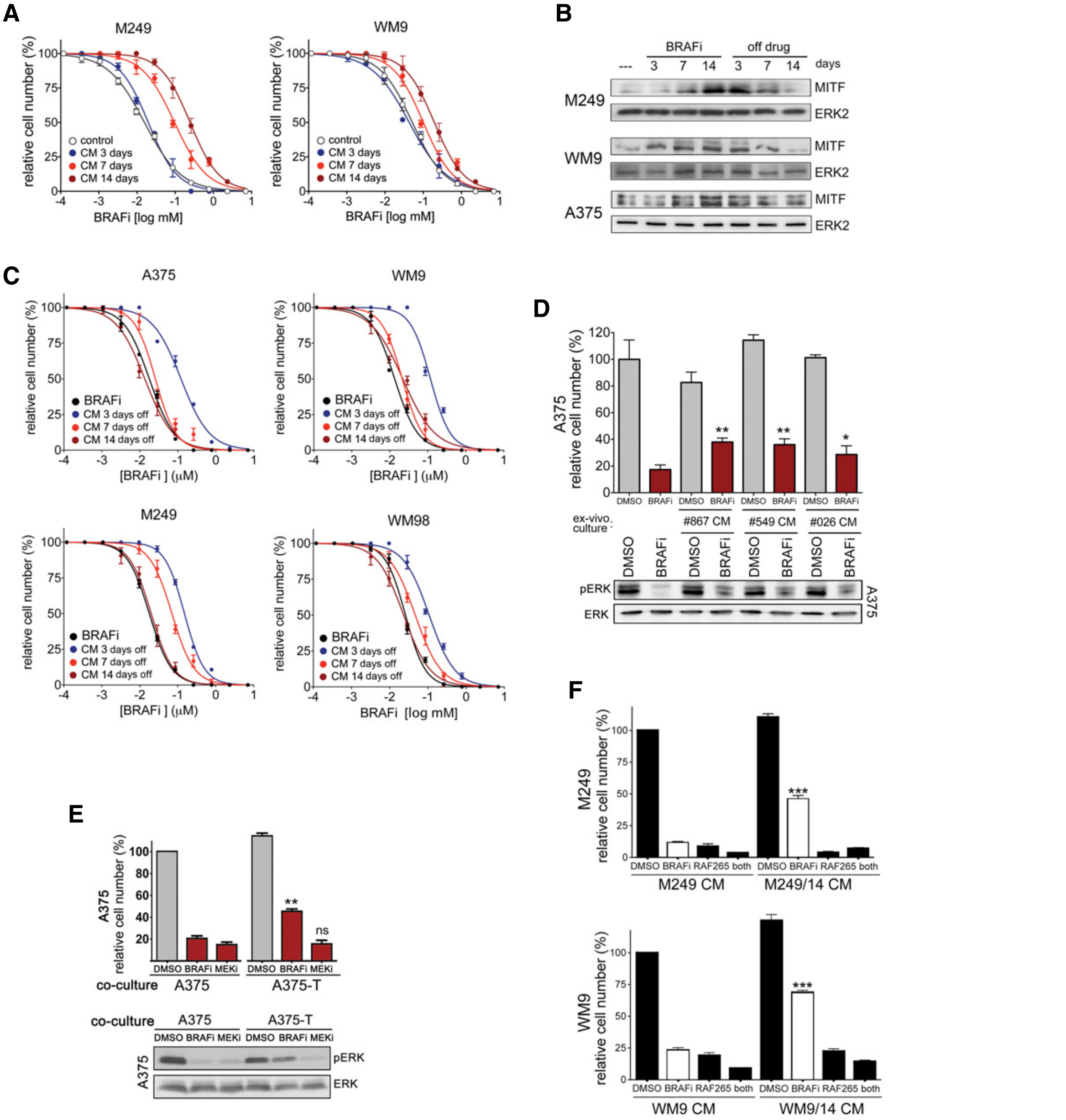

Figure EV3. Long-term BRAFi treatment produces drug tolerance.

A Dose–response curves for vemurafenib (BRAFi) for the indicated cell lines. The cell lines were treated in either DMEM (control) or conditioned medium, which was derived from cells treated with vemurafenib for the indicated times.

B Western blot for MITF in the indicated cell lines treated with vemurafenib (BRAFi) for the indicated times.

C Dose–response curves for vemurafenib (BRAFi) for the indicated cell lines. The cell lines were treated in either DMEM (control) or conditioned medium, which was derived from cells treated with vemurafenib, and then, the inhibitor was removed for the indicated times.

D Quantification of relative cell numbers. A375 cells were either left untreated or were treated with 0.5 μM vemurafenib (BRAFi) in the presence of conditioned medium from *ex vivo* cultures. DMSO-treated A375 cells were set at 100%. A Western blot for pERK and ERK under the respective conditions is shown. *P*: probability by one-way ANOVA (with Dunnett's *post hoc* test); $**P = 0.0014$ (#867), $**P = 0.0026$ (#549) and $*P = 0.0387$ (#026).

E Western blots of A375 cells for pERK and total ERK, and quantification of relative cell number of A375 cells when co-cultured with either A375 or with A375-T cells. Cells were treated with DMSO or vemurafenib (BRAFi) or selumetinib (MEKi) for 48 h. A375 cells in the presence of A375 cells were set at 100%. *P*: probability by one-way ANOVA (with Tukey's *post hoc* test); ns $P > 0.05$, $**P = 0.001$.

F Quantification of relative cell number of the indicated cell lines treated with DMSO or with vemurafenib (BRAFi). During drug treatment, cells were incubated with conditioned medium derived from untreated cells or cells pre-treated with BRAFi for 14 days. *P*: probability by one-way ANOVA (with Tukey's *post hoc* test); $***P < 0.0001$.

Data information: Data are pooled from three independent experiments and shown as mean ± SEM.

 © 2017 The Authors

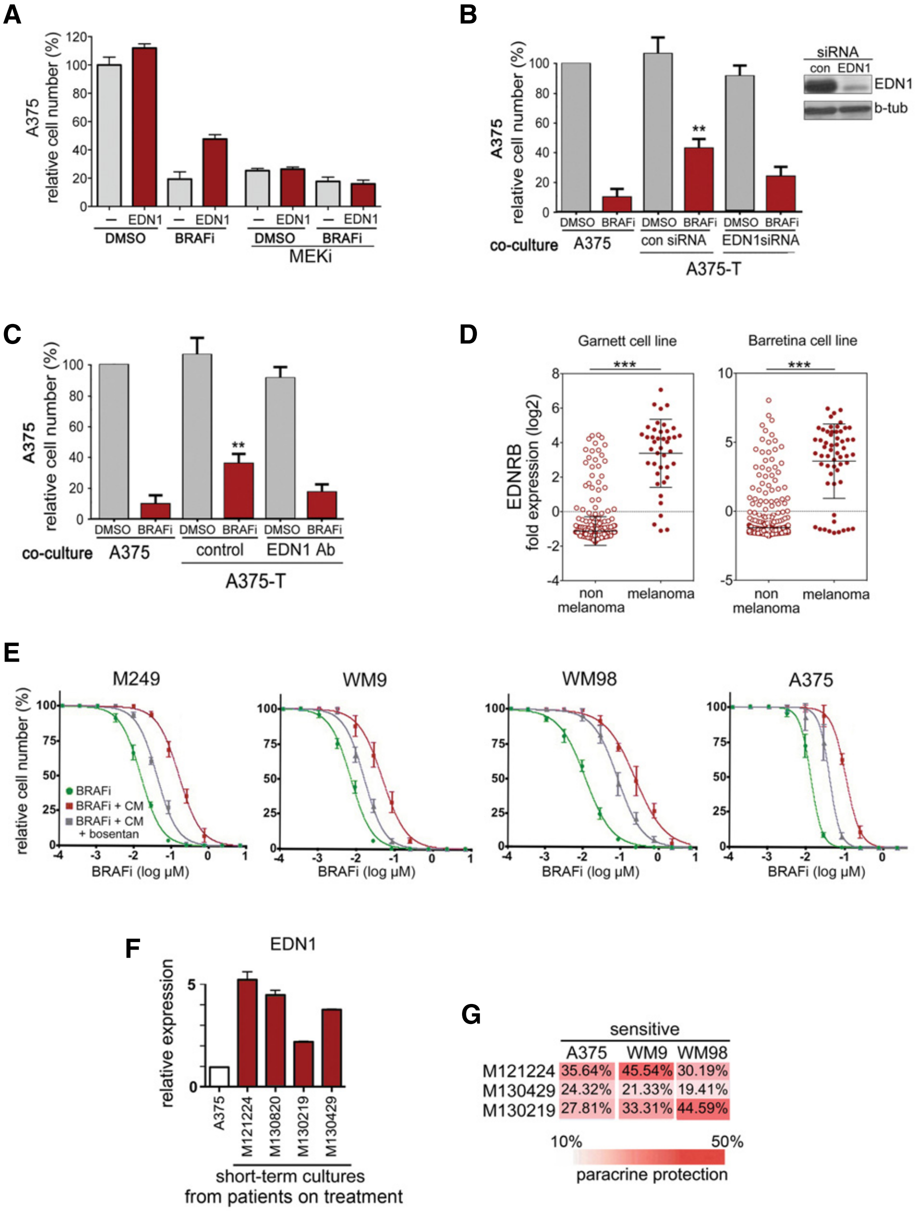

Figure EV4. EDN1 and EDNRB are involved in paracrine protection.

A Quantification of relative cell number of A375 cells when treated with EDN1 in the absence or presence of vemurafenib (BRAFi) and selumetinib (MEKi).

B Quantification of relative cell number of A375 cells when co-cultured with A375-T cells treated with a control or an EDN1 specific siRNA. *P*: probability by one-way ANOVA (with Tukey's *post hoc* test); $^{**}P = 0.0011$. A Western blot demonstrating the degree of EDN1 knock down in A375-T cells is shown. Beta-tubulin served as loading control.

C Quantification of relative cell number of A375 cells when co-cultured with A375-T cells in the presence of a control or an EDN1-specific blocking antibody. *P*: probability by one-way ANOVA (with Tukey's *post hoc* test); $^{**}P = 0.0016$.

D The indicated cancer cell line datasets were analysed for EDNRB expression using Oncomine. *P*: probability by *t*-test; $^{***}P < 0.0001$ (Garnett and Barretina). Mean ± SD; $n = 732$ (Garnett); $n = 917$ (Barretina).

E BRAFi dose–response curves for vemurafenib (BRAFi) or the indicated cell lines. The cell lines were treated in DMEM (control) or in conditioned medium derived from cells treated for 14 days with BRAFi in the absence or presence of bosentan.

F Real-time qPCR of EDN1 in short-term cultures from progressed patients.

G Analysis of paracrine protection in a panel of short-term cultures from progressed patients and drug-sensitive melanoma cells. Drug-sensitive A375, WM9 or WM98 cells were co-cultured with the indicated melanoma cell cultures, and paracrine detection was determined as indicated. Data are the mean of three independent experiments.

Data information: Data for (A–C, E and F) are pooled from three independent experiments and presented as mean ± SEM.

Figure EV5. EDNRA and EDNRB are expressed in AXL-high and MITF-high cells and contribute to paracrine protection.

A Pearson correlation of expression (log2) of EDNRA with AXL in melanoma cell lines from the Barretina and Garnett (Garnett *et al*, 2012) datasets deposited in Oncomine.

B Pearson correlation of expression (log2) of EDNRB with either the expression (log2) of EDNRA or AXL in melanoma cell lines from the Barretina dataset (Barretina *et al*, 2012) deposited in Oncomine.

C Analysis of cells in S-phase. The indicated cell lines were treated with 0.5 μM vemurafenib (BRAFi) and bosentan either alone or in combination and in the presence of conditioned medium from A375-T cells for 24 h, and 4 h before analysis EdU was added to the cultures. *P*: probability by *t*-test; ***$P < 0.0001$ (A375), ***$P < 0.0001$ (WM164), ***$P < 0.0001$ (WM793) and *$P = 0.0445$ (RPMI7951).

D Western blot for pERK and ERK of A375 and WM793 cells treated with vemurafenib (BRAFi) and bosentan either alone or in combination and in the presence of conditioned medium.

E Quantification of relative cell number of A375 cells when co-cultured with either A375 or with A375-T cells in the absence or presence of vemurafenib (BRAFi), alone or in combination with BQ788 or BQ123. *P*: probability by one-way ANOVA (with Dunnett's *post hoc* test); ns $P > 0.05$, ***$P < 0.0001$.

F Quantification of relative cell number of A375 and WM793 cells treated with vemurafenib (BRAFi) and macitentan or BQ788 or BQ123 either alone or in combination and in the presence of conditioned medium. *P*: probability by one-way ANOVA (with Dunnett's *post hoc* test); ns $P > 0.05$, ***$P < 0.0001$ (WM793 BQ123 + BRAFi, A375 Mac + BRAFi, BQ788 + BRAFi), ***$P = 0.0004$ (WM793 Mac + BRAFi).

G Quantification of relative cell number of WM793 cells when co-cultured with A375-T cells in the presence of a control or an EDN1-specific blocking antibody. Conditioned medium from A375 cells was used as control. ns $P > 0.05$, ***$P = 0.0005$; Tukey's test.

H Quantification of relative cell number of WM793 cells treated with EDN1 in the absence or presence of vemurafenib (BRAFi), alone or in combination with RAF265 or dovitinib (RTKi) or GO-6983 (PKCi). *P*: probability by one-way ANOVA (with Tukey's *post hoc* test); ns $P > 0.05$, ***$P < 0.0001$ (RAF265 + BRAFi, PKCi + BRAFi).

I Western blot for pERK and ERK2 of WM793 cells when treated with EDN1 in the presence of vemurafenib (BRAFi) and RAF265, dovitinib (RTKi) or GO-6983 (PKCi).

Data information: Data are pooled from three independent experiments and presented as mean ± SEM.

 © 2017 The Authors

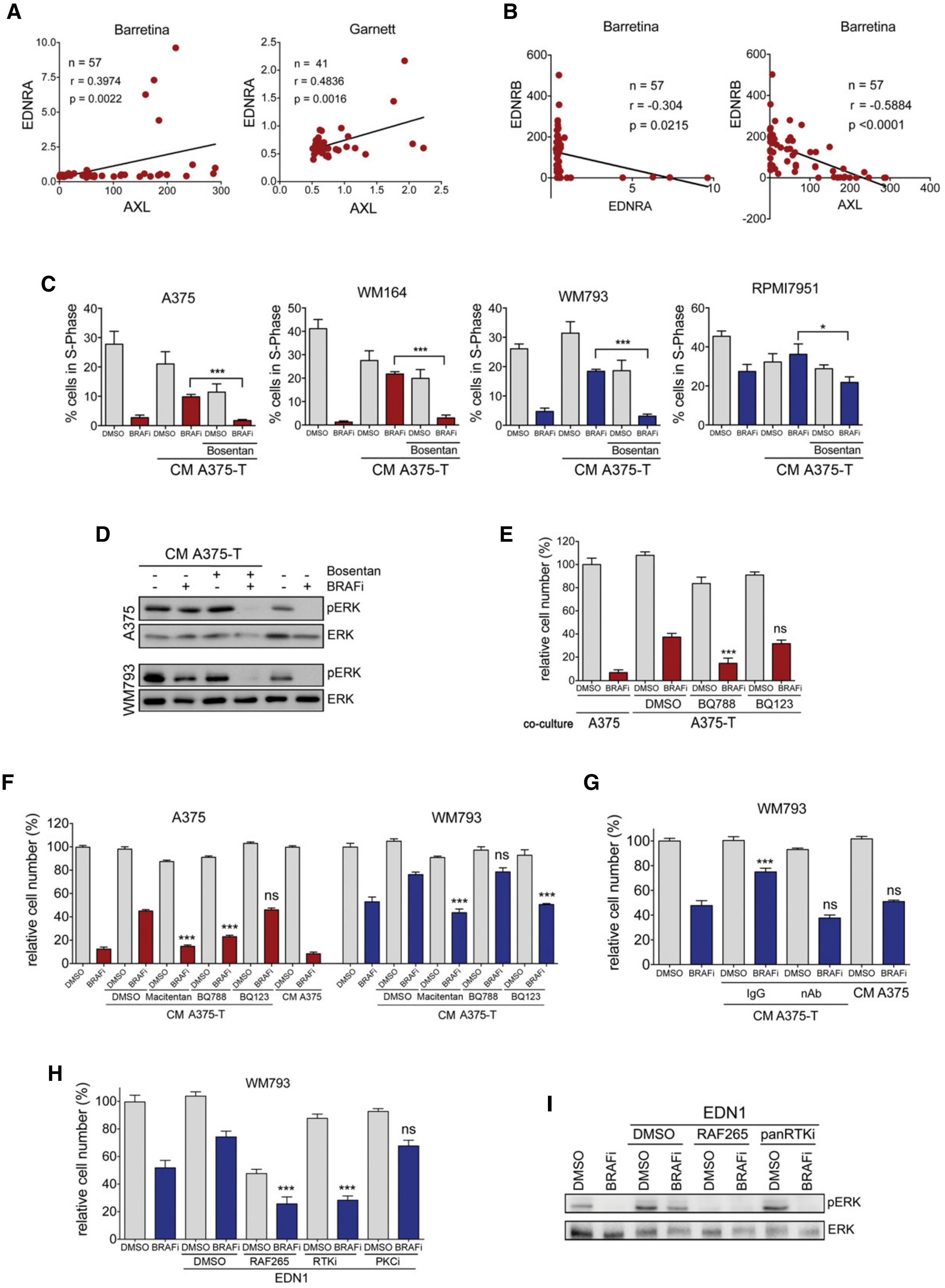
A
Barretina
n = 57
r = 0.3974
p = 0.0022
EDNRA
AXL
Garnett
n = 41
r = 0.4836
p = 0.0016
B
Barretina
EDNRB
EDNRA
n = 57
r = -0.304
p = 0.0215
n = 57
r = -0.5884
p <0.0001
C
A375
WM164
WM793
RPMI7951
% cells in S-Phase
DMSO BRAFi DMSO BRAFi DMSO BRAFi
Bosentan
CM A375-T
D
CM A375-T
Bosentan
BRAFi
pERK
ERK
A375
WM793
E
relative cell number (%)
co-culture
A375
A375-T
DMSO
BQ788
BQ123
ns
F
A375
WM793
Macitentan
CM A375
G
IgG
nAb
H
EDN1
RAF265
RTKi
PKCi
I
panRTKi

Figure EV5.

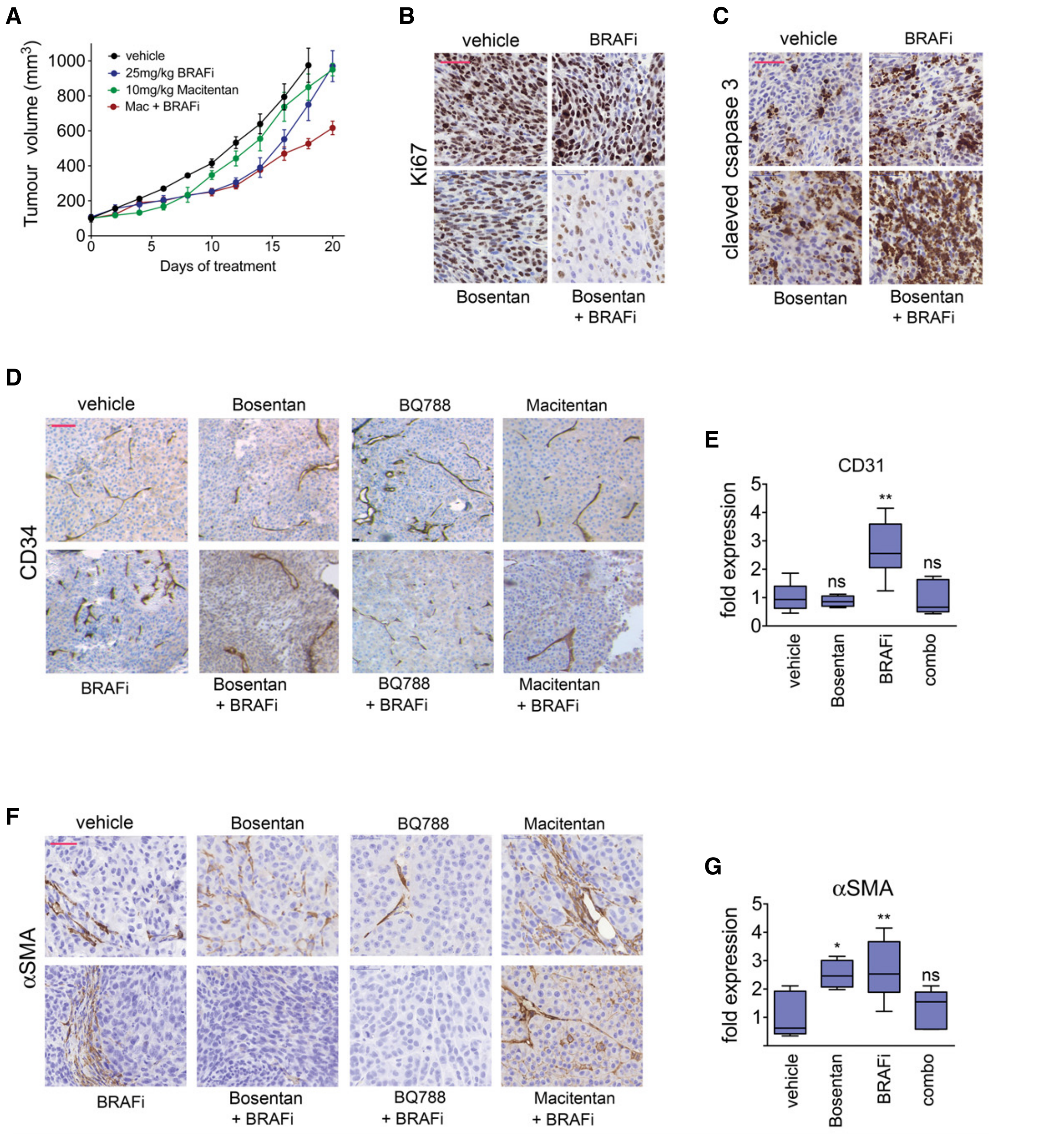

Figure EV6. EDNRA and EDNRB antagonists impact on melanoma cells and the tumour microenvironment.

A Nude mice (n = 5–6 mice per group) bearing A375 tumours were treated with vehicle, vemurafenib (25 mg/kg/qd) or macitentan (10 mg/kg/qd) alone or in combination for 20 days.

B IHC for Ki67 in A375 xenografts from mice treated as indicated. Scale bar: 50 μm.

C IHC for cleaved caspase-3 in A375 xenografts from mice treated as indicated. Scale bar: 50 μm.

D IHC for cleaved CD34 in A375 xenografts from mice treated as indicated. Scale bar: 100 μm.

E qRT–PCR for CD31 from tumours treated with BRAF inhibitor or bosentan alone or in combination. P: probability by one-way ANOVA (with Dunnett's *post hoc* test); ns $P > 0.05$, **P = 0.0012. Error bars, min and max values; box limits, second and third quartiles; horizontal line, median.

F IHC for cleaved αSMA in A375 xenografts from mice treated as indicated. Scale bar: 50 μm.

G qRT–PCR for αSMA from tumours treated with BRAF inhibitor or bosentan alone or in combination. P: probability by one-way ANOVA (with Dunnett's *post hoc* test); ns $P > 0.05$, *P = 0.0255 (Bosentan) and **P = 0.0060 (BRAFi). Error bars, min and max values; box limits, second and third quartiles; horizontal line, median.

Data information: Data are from n = 5–6 mice per group and are presented as mean tumour volumes ± SEM.

 © 2017 The Authors
