# Supplementary figures and images for: Targeting endothelin receptor signalling overcomes heterogeneity driven therapy failure
Source: EMBO Mol Med. 2017 Jun 12;9(8):1011–29. doi: 10.15252/emmm.201607156 (PMC5538298; doi:10.15252/emmm.201607156)

**Source Data.** Immunoblots from Figure 1  
Dashed outline indicates blot area presented in figure.

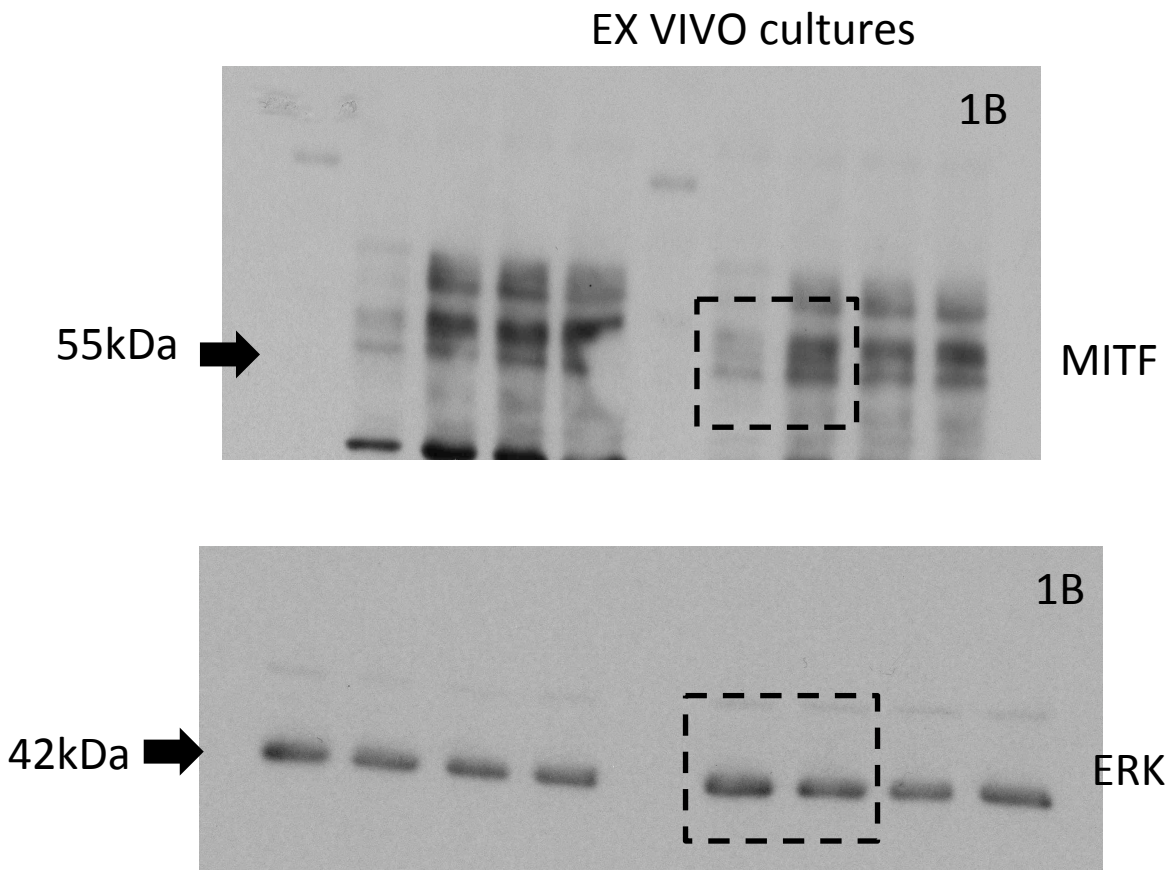

Supplement: Supplementary file 3 — Source Data for Figure 1 [file EMMM-9-1011-s003.pdf]

**Source Data.** Immunoblots from Figure 2  
Dashed outline indicates blot area presented in figure.

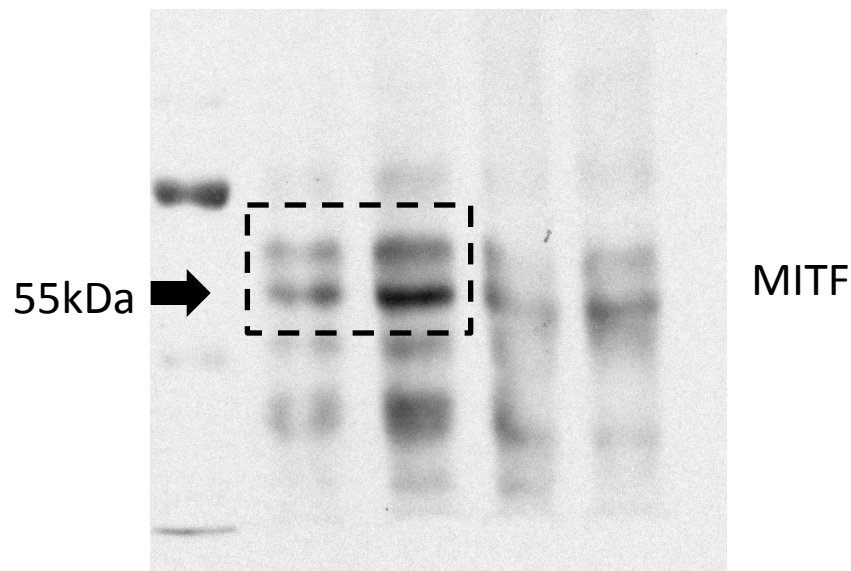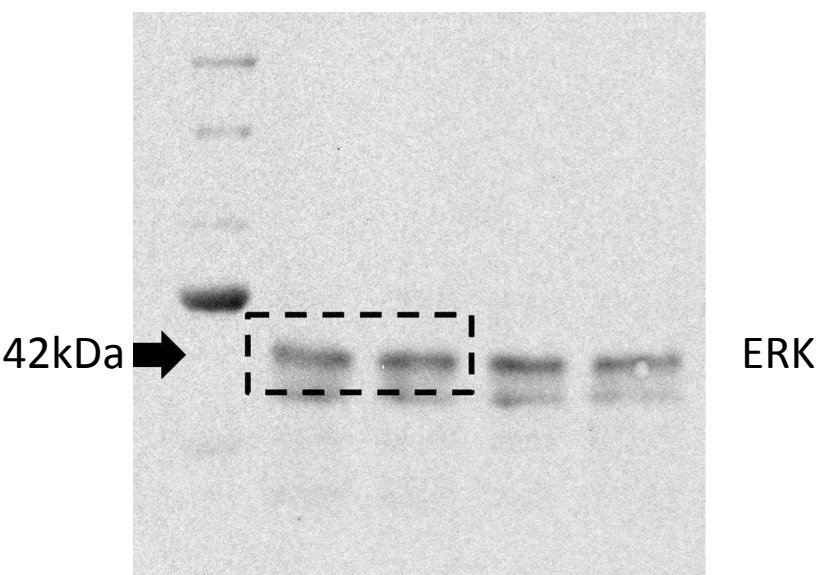

Supplement: Supplementary file 4 — Source Data for Figure 2 [file EMMM-9-1011-s004.pdf]

**Source Data.** Immunoblots from Figure 4  
Dashed outline indicates blot area presented in figure.

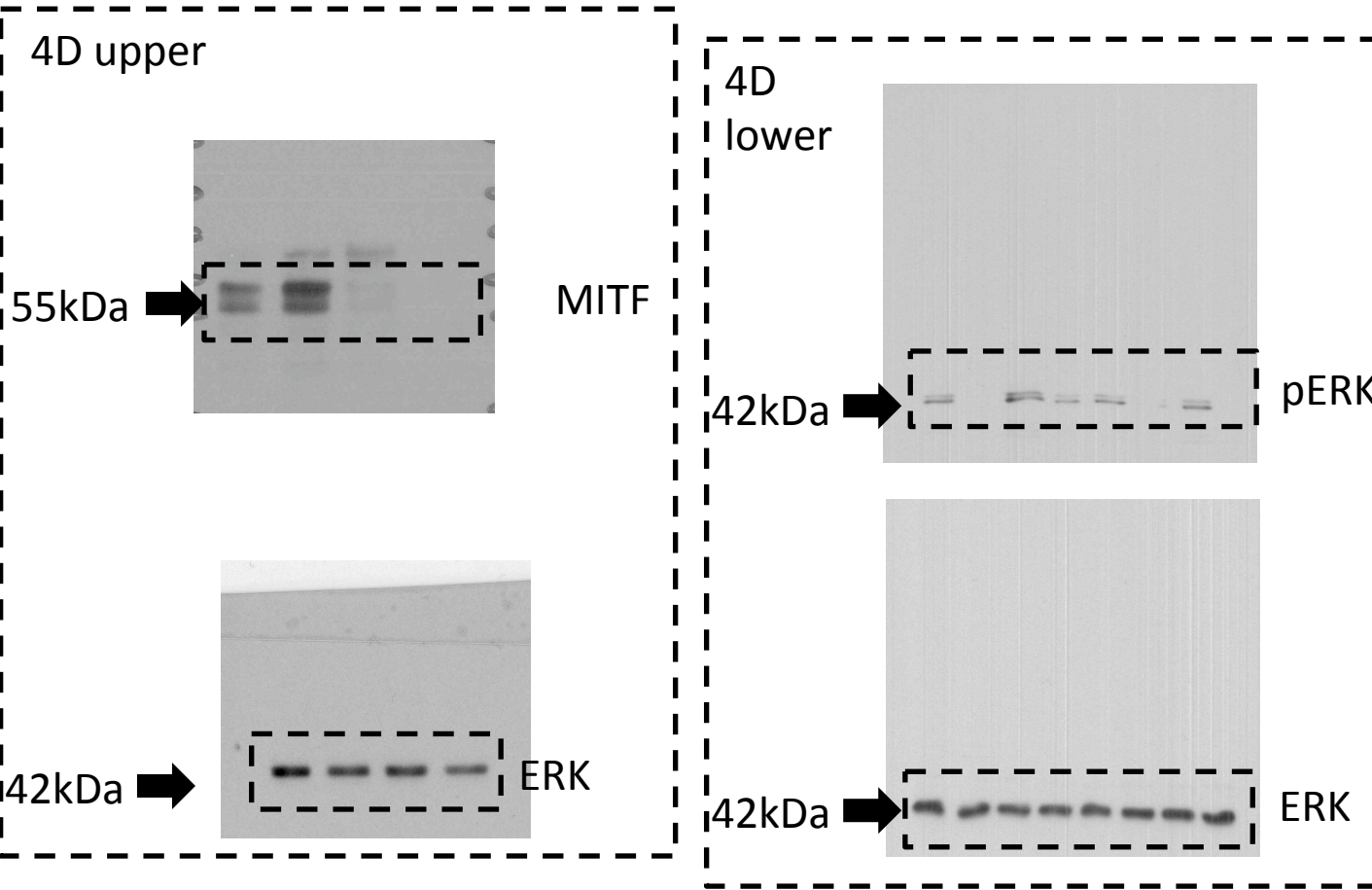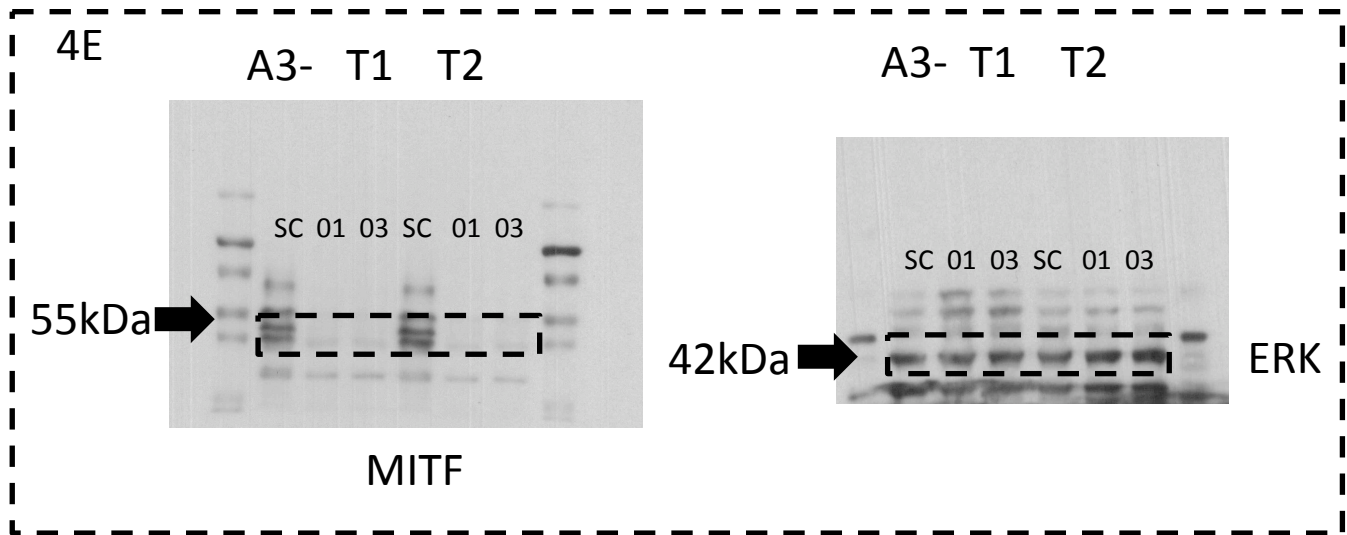

Supplement: Supplementary file 6 — Source Data for Figure 4 [file EMMM-9-1011-s006.pdf]

**Source Data.** Immunoblots from Figure 7  
Dashed outline indicates blot area presented in figure.

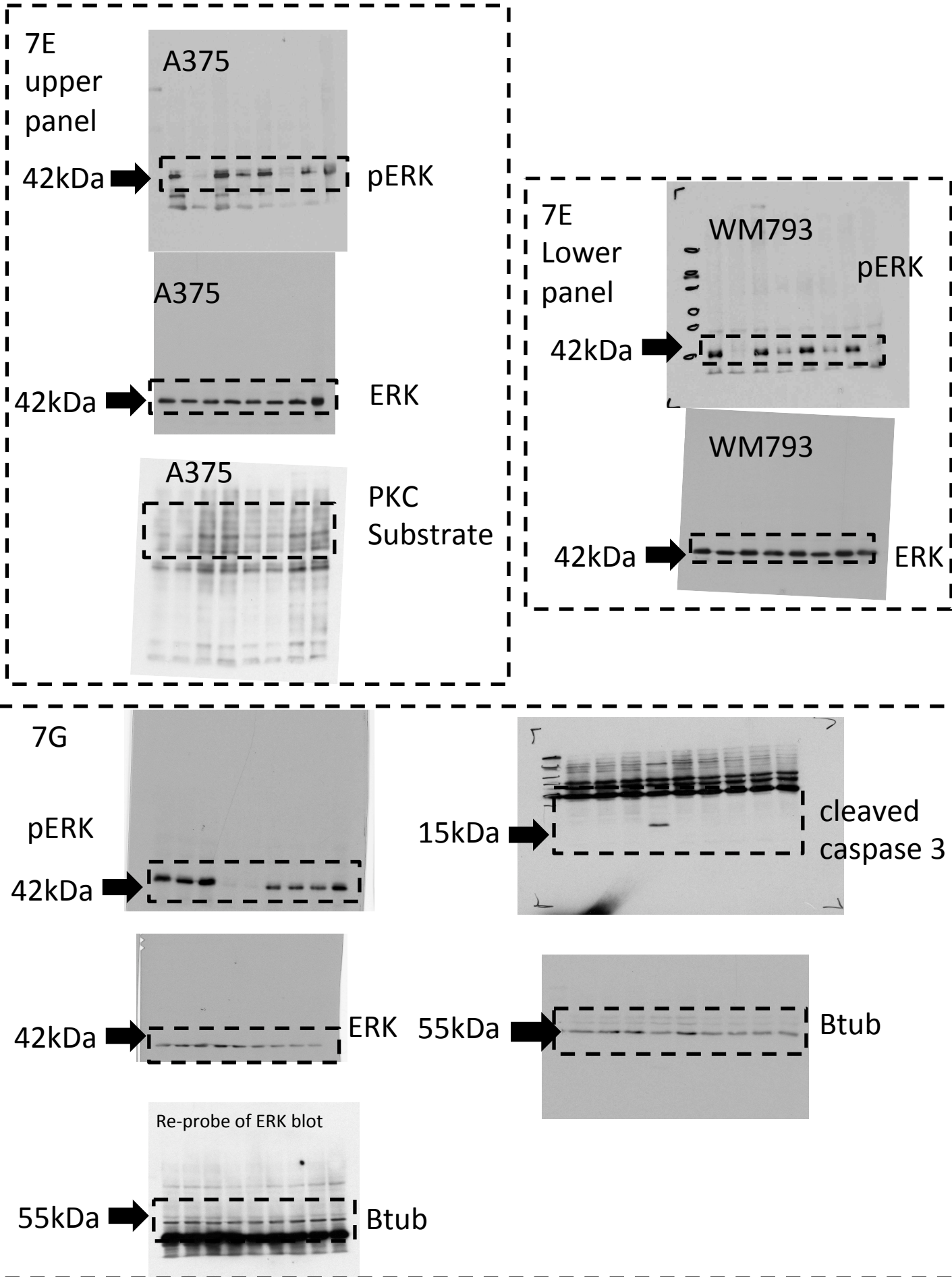

Supplement: Supplementary file 8 — Source Data for Figure 7 [file EMMM-9-1011-s008.pdf]
