# Supplementary material for: Targeting endothelin receptor signalling overcomes heterogeneity driven therapy failure
Source: EMBO Mol Med. 2017 Jun 12;9(8):1011–29. doi: 10.15252/emmm.201607156 (PMC5538298; doi:10.15252/emmm.201607156)

Source Data. Immunoblots from Figure 3
Dashed outline indicates blot area presented in figure**.**

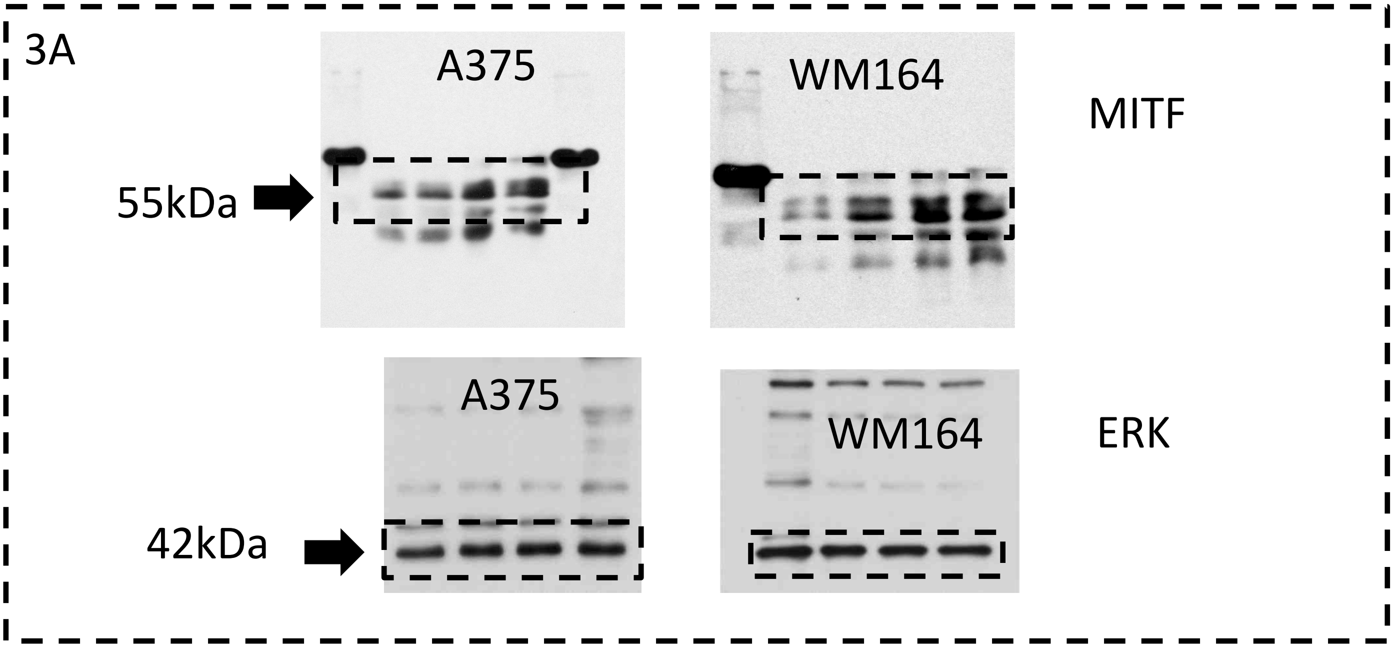

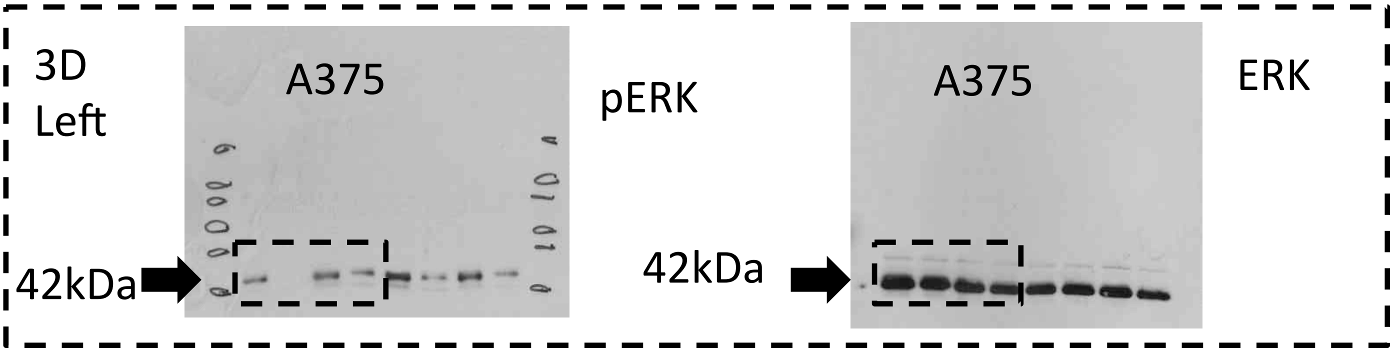

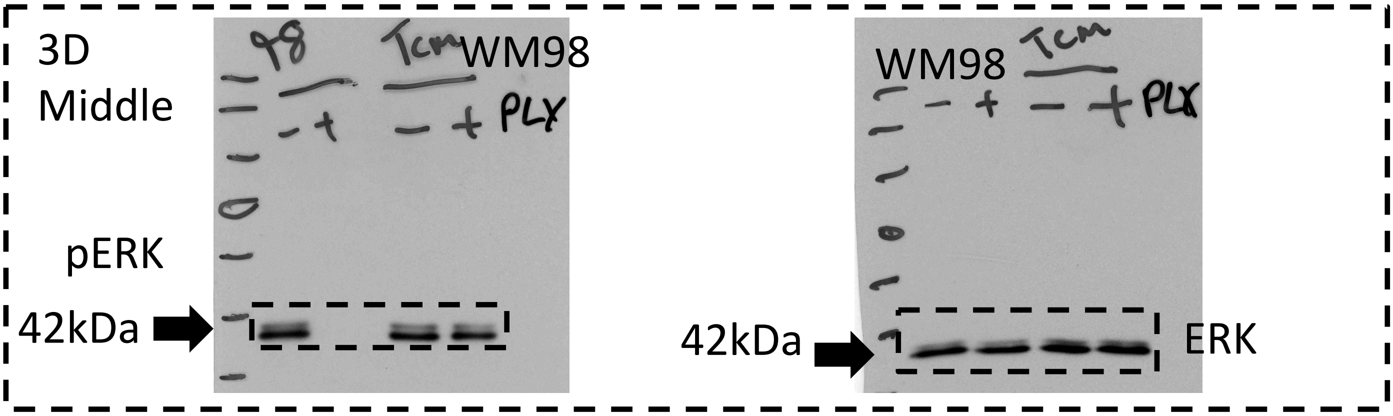

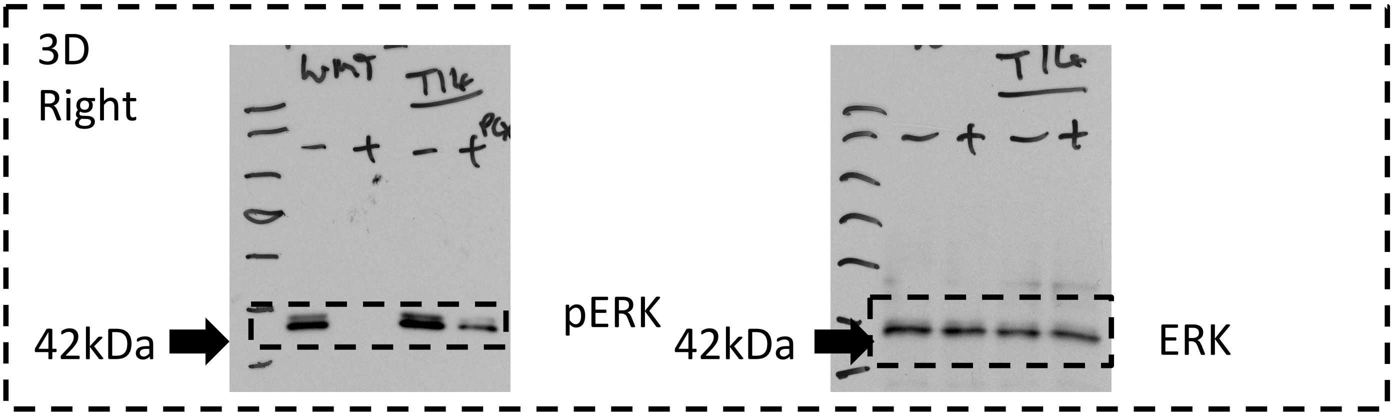

Source Data. Immunoblots from Figure 3 continued
Dashed outline indicates blot area presented in figure.

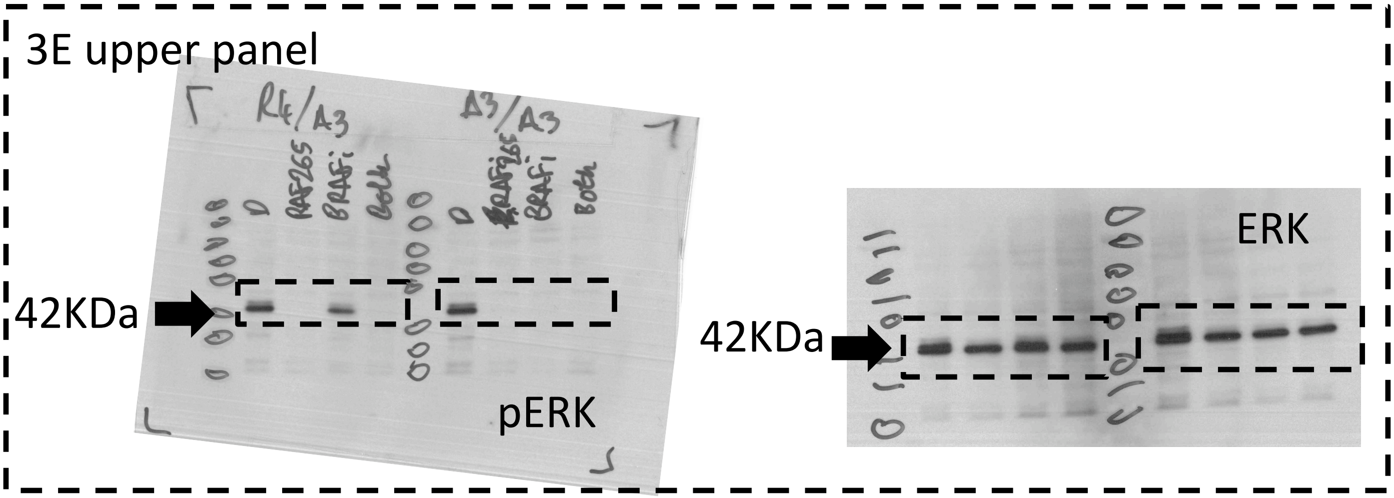

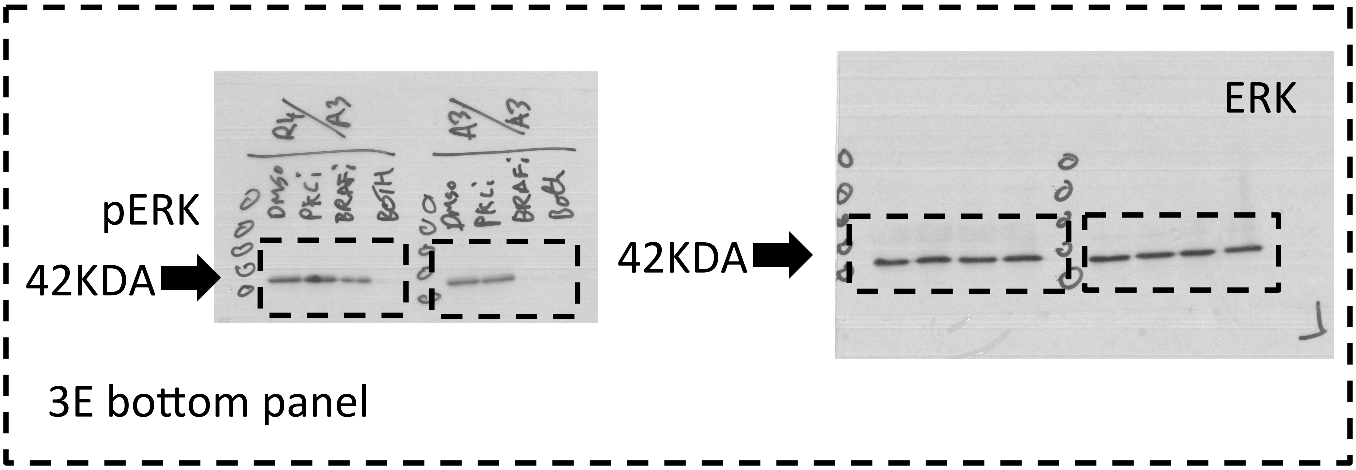

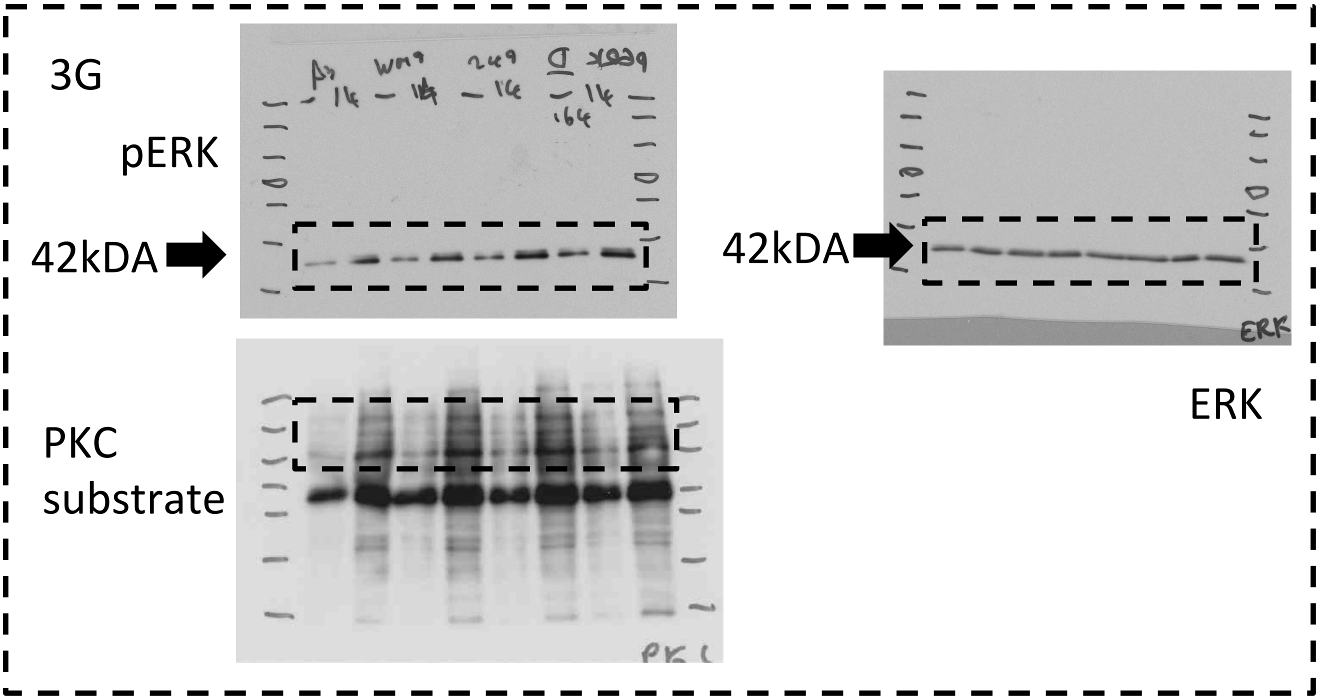

Supplement: Supplementary file 5 — Source Data for Figure 3 [file EMMM-9-1011-s005.pdf]
