# Supplementary material for: Targeting endothelin receptor signalling overcomes heterogeneity driven therapy failure
Source: EMBO Mol Med. 2017 Jun 12;9(8):1011–29. doi: 10.15252/emmm.201607156 (PMC5538298; doi:10.15252/emmm.201607156)

Source Data. Immunoblots from Figure 5
Dashed outline indicates blot area presented in figure.

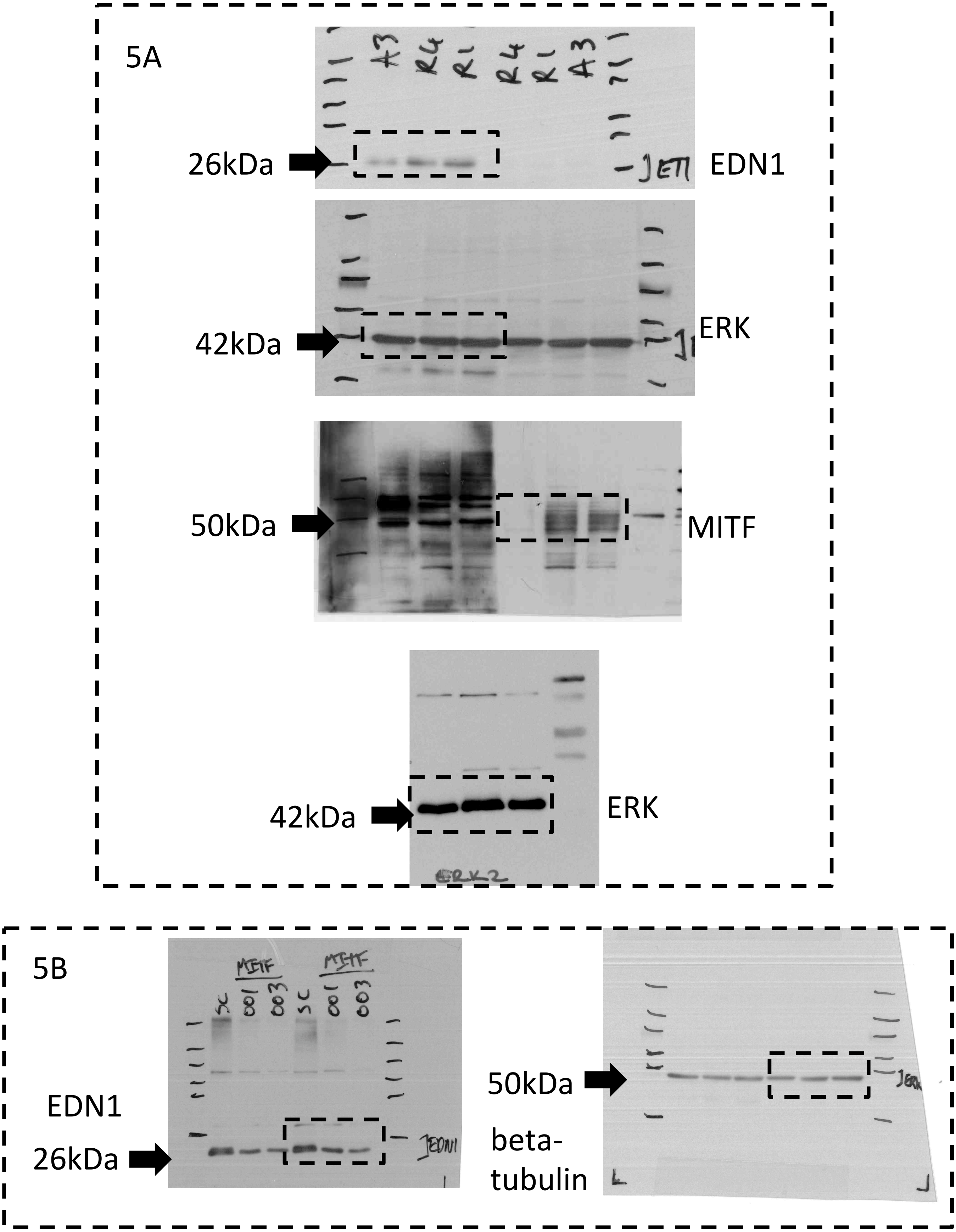

Source Data. Immunoblots from Figure 5 continued
Dashed outline indicates blot area presented in figure.

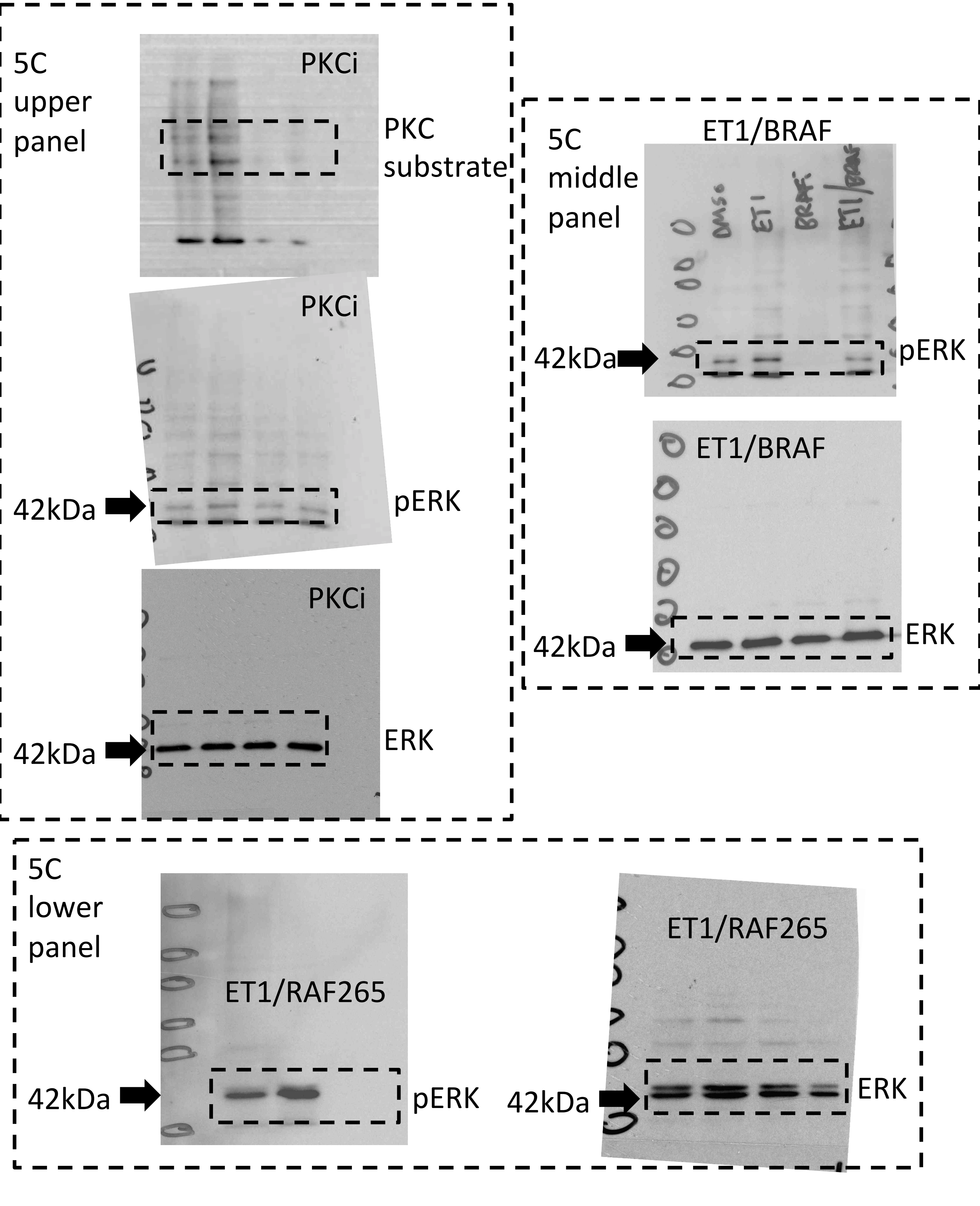

Supplement: Supplementary file 7 — Source Data for Figure 5 [file EMMM-9-1011-s007.pdf]
